# Sexual and reproductive health knowledge, attitudes, and behaviors: A survey of 12–17 year old Sudanese refugees in Chad

**DOI:** 10.1371/journal.pgph.0002597

**Published:** 2024-01-25

**Authors:** Theodora Varelis, Vincent de Paul Allambademel, Alexis Ngarmbatedjimal, Alladoum Ndingayande, Aminata Diarra, Katchebe Vourbane, Robert Madjigoto, Samy Luketa, Sara E. Casey

**Affiliations:** 1 RAISE Initiative, Heilbrunn Department of Population and Family Health, Mailman School of Public Health, Columbia University, New York, NY, United States of America; 2 Laboratoire de Sociologie, d’Anthropologie et des Etudes Africaines (LASA), Department of Sociology, College of Humanities and Social Sciences, University of N’Djamena, N’Djamena, Chad; 3 International Rescue Committee, N’Djamena, Chad; Johns Hopkins University Bloomberg School of Public Health, UNITED STATES

## Abstract

Adolescents living in humanitarian settings are often at a higher risk of negative sexual and reproductive health outcomes, and yet, limited information is available on adolescents’ experiences and needs in these settings while available services do not always correspond to their needs. This study explored knowledge, attitudes, and behaviors relating to sexual and reproductive health among 12–17 year old refugees from Darfur currently living in two refugee camps in eastern Chad. The research team conducted a cross-sectional survey of 689 adolescent girls and boys, informed by participatory research activities, to explore key sexual and reproductive health topics. This study found that sexual and reproductive health knowledge among adolescents is low, with only 69.1% able to identify at least one modern contraceptive method. Early marriage was uncommon (5% of girls, 0.8% of boys), but 17.6% of adolescents had already had a romantic relationship. Few adolescents (11.4%) had ever had sex, but among these adolescents,18.4% reported using a condom the last time they had sex. No boys reported current modern contraceptive use, but 28.3% of girls, both married and unmarried, reported current use. These findings demonstrate the importance of making sexual and reproductive health services in humanitarian settings more adolescent-responsive and minimizing barriers to access, including addressing community stigma surrounding adolescents’ use of sexual and reproductive health services and increasing trust in the confidentiality of sexual and reproductive health services in the camps.

## Introduction

### Background

Adolescence is a period of transition between childhood and adulthood, typically between ages 10 and 19 [1]. During this window of time, adolescents seek more independence, including exploring and adopting new health-related behaviors. As such, this period presents an opportunity for developing positive practices that are essential for future good health and well-being. Despite the evidence that health-related behaviors in adolescence can have major impacts across the lifecourse, adolescents’ sexual and reproductive health (SRH) needs remain largely unmet globally [2, 3]. As of 2019, an estimated 32 million adolescent girls aged 15–19 were married or sexually active and did not want to become pregnant in the next two years, but only 43% of them, or approximately 14 million girls, were not using a modern contraceptive method [4].

Access to SRH services is even more difficult for adolescents living in humanitarian settings where services may not correspond to adolescents’ specific needs and where they continue to be overlooked [5, 6]. Crises typically cause a disruption in livelihoods, support systems, and social networks, which may force adolescents to take on adult responsibilities or families to adopt negative coping mechanisms. For example, adolescents affected by displacement may seek paid work outside the home, exchange sex for money or other needed items, or be given in marriage at younger ages to decrease their family’s financial burdens [5, 7]. In these situations, adolescents are at increased risk of sexual exploitation or coercion, sexual violence, sexually transmitted infections, unintended pregnancy, unsafe abortions, and other health consequences, all of which can have lifelong, negative impacts on their health and life trajectory.

In addition, crises disrupt and eventually deteriorate health systems, often resulting in the lack of appropriate services, low-quality services, or stockouts of essential medicines and equipment. Gendered attitudes and beliefs about the appropriateness of SRH services for adolescents can strongly influence adolescents’ access to SRH information and services, even when they are available [8].

While the *Adolescent Sexual and Reproductive Health Toolkit for Humanitarian Settings* [5], and the *Inter-Agency Field Manual on Reproductive Health in Humanitarian Settings* [8] provide guidance on delivering SRH services to adolescents in humanitarian settings, limited information is available about displaced adolescents’ knowledge, attitudes, and behaviors relating to SRH [6]. More robust evidence is needed to understand the SRH needs specific to adolescents living in humanitarian settings.

### Context

Evidence on adolescent SRH in the Sahel region is lacking. Eastern Chad hosts thousands of Sudanese refugees, most of whom fled Sudan during the Darfur crisis in 2003 [9]. This conflict involved systematic targeting of civilians, including a scorched earth policy and sexual violence [10]. The resurgence of violence in Sudan in April 2023 drove an additional 300,000 Sudanese refugees into Chad in the first three months of the crisis [11]. Prior to this renewed violence, an estimated 52,135 Sudanese refugees lived in Mile and Kounoungou refugee camps in Wadi Fira province [12].

Although little data on adolescent refugees from Darfur are available, key adolescent SRH indicators are generally poor in Chad. For example, the median age at first sex in Wadi Fira province, where the study took place, is 16.7 for women aged 20–49 [13]. The adolescent (15–19 years) birth rate is 109 per 1000 women in Wadi Fira and 117 for West Darfur, both of which are much higher than the 2021 global adolescent birth rate of 42.5 [14–16]. Condom use remains low as well: only 7.5% of sexually active girls aged 15–24 in Chad reported using a condom the last time they had sex with a non-marital sexual partner in the last 12 months [14].

The International Rescue Committee (IRC), in collaboration with the Ministry of Public Health and Association Tchadienne pour le Bien-Etre Familial (ASTBEF), the local member agency of the International Planned Parenthood Federation, launched the Protection, Gender and Health (ProGeSan) program in 2020 to improve the social empowerment of women in 15 health centers in three districts of Guera province and four refugee camps in Wadi Fira province through improved access to maternal, newborn, infant, adolescent and sexual and reproductive health services and response to gender-based violence. The RAISE Initiative at Columbia University Mailman School of Public Health, which leads an action research component of ProGeSan with the University of N’Djamena, has worked with IRC and partners in Chad to identify and address challenges to improve SRH services since 2009. IRC began supporting health services for refugees and host communities in eastern Chad in 2004, with a specific focus on SRH since 2009. IRC has supported the health centers in the two camps where this study was conducted since 2017 with maternity care, including antenatal and delivery care, and a range of short- and long-acting contraceptive methods. They support three midwives at each health center who also received training on adolescent SRH in addition to other SRH topics. In response to low SRH service utilization by adolescents, IRC Chad, the University of N’Djamena, and RAISE conducted a study in Mile and Kounoungou refugee camps to explore adolescent refugees’ SRH knowledge, attitudes, and behaviors in 2022. This manuscript presents findings from 12–17 year old girls and boys.

## Methods

We conducted a representative cross-sectional survey, informed by participatory research activities, with adolescents living in Kounoungou and Mile refugee camps in Wadi Fira.

### Participatory research activities

Prior to the survey, participatory research activities were held with groups of 12–18 adolescents stratified by sex and age (12–14 years, 15–19 years). Participants meeting the age and sex criteria were recruited purposively by community health workers and youth group representatives, with at least one adolescent from each of the 12 zones in Mile and the nine zones in Kounoungou. Four bilingual French-Arabic facilitators (two female, two male), recruited from the local host community, participated in a five-day training on adolescent SRH and participatory research methodology, including review of a detailed facilitator’s guide adapted from guides previously used by IRC in other countries, and practice activities.

The participatory research consisted of a day-long session facilitated in Arabic by the trained team members, with support from French-speaking research team members from both universities who were observing with a local translator. These sessions included the following activities: discussing and defining key SRH terms, creating social and developmental timelines for girls and boys, completing spider diagrams identifying and prioritizing adolescents’ priority needs to maintain good SRH, and discussions around barriers and enablers of their access to SRH services. Adolescents worked on each activity in small groups of 4–5 to draw their responses on flipchart paper, followed by large group discussion reflecting together on the activity. Both small and large group discussions were recorded, and audio recordings for selected activities were transcribed and translated from Arabic into French. At the end of each day, the facilitators added notes to the posters created by adolescents during the activities and translated any text into French; the posters were also photographed for storage. Information from the activities was documented in recording books for compilation and analysis. These sessions took place 21–24 June 2022.

### Survey design

We conducted a representative survey of adolescent girls (age 12–19 years) and boys (12–17 years). Boys aged 18–19 were excluded because older boys often leave the camps for work and would therefore be difficult to find in the camps. This analysis also excludes 18–19 year old girls. The sampling frame was drawn from household lists stratified by age and sex maintained by UNHCR. In each camp, 150 girls were randomly selected from each of three age groups (12–14, 15–17, 18–19), and 75 boys from each of two age groups (12–14 and 15–17). Community health workers (CHWs) and youth group representatives in each camp reviewed the lists to indicate which adolescents were known to be not currently living in the camp. An additional 60–70 girls per age group and 40–45 boys per age group in each camp were then randomly selected to ensure achievement of the total desired sample size of 100 girls per age group per camp and 50 boys per age group per camp.

### Survey procedures

Separate but similar questionnaires were developed in French for girls and boys, adapted from multiple sources, including *Demographic and Health Surveys*, *Gender and Adolescence*: *Global Evidence (GAGE)*, *World Health Organization* and tools previously used by RAISE and IRC. The questionnaires covered knowledge and attitudes towards SRH, marriage, sexual activity, experience with pregnancy and delivery, contraception, and safety and sexual violence. The French questionnaire was programmed onto tablets using KoboToolbox.

Female and male bilingual French-Arabic interviewers with survey experience recruited from the host community discussed how to translate the questions into local Arabic during an 8-day training. The training also covered SRH terminology, values clarification activities and survey techniques, including the importance of confidentiality and consent procedures. Interviewers participated in practical exercises to assure mastery of material. Three teams of four female interviewers and one team of five male interviewers conducted the survey. The teams worked with CHWs and youth group representatives to locate selected adolescents at home. When the adolescent was temporarily absent, efforts were made to return on subsequent days to complete the interview. If an individual could not be found, they were replaced with a randomly selected peer in the same age cohort who lived in the same or a nearby zone of the camp. Once an adolescent agreed to participate, they were interviewed in Arabic in a private location near their home. Data collection took place 5–20 July 2022.

### Data analysis

Each evening, data were uploaded from the tablets to the KoboToolbox server for review. Data were downloaded from KoboToolbox into Microsoft Excel and subsequently exported to SPSS (v28) for cleaning and analysis. Data were stratified by sex; girls 18–19 years (n = 220) were excluded from this analysis to allow comparability of girls and boys. Chi-square (categorical variables) were used to describe and compare results between girls and boys aged 12–17; p-values < .05 were considered significant differences. Observations with missing data for specific variables were excluded from analysis of those variables; variables with ≥5% missing data are indicated in the tables. Modern contraceptive methods were defined as tubal ligation, vasectomy IUDs, implants, injectables, oral contraceptive pills, emergency contraception and male or female condoms. Contraceptive knowledge was defined as spontaneous or prompted (have you ever heard of the method) knowledge of any modern method.

The research team together reviewed the digitized posters and transcripts of selected discussions during participatory research and conducted a thematic synthesis of the discussions and posters. The themes from the discussions and results of participatory activities that contribute to understanding of the survey findings are presented below to complement the survey results.

### Ethical considerations

Ethical approvals for all study components and consent procedures were obtained from the Institutional Review Board of Columbia University and the Direction de la Recherche et de l’Innovation du Ministère de l’Enseignement Supérieur, de la Recherche et de l’Innovation in Chad. Parental consent for 15–17 year olds and written documentation of consent for all participants were waived as this study met the criteria for minimal risk and a written consent document would be the only record linking the participant to the study. Verbal parental consent was obtained for adolescents 12–14 years, and these younger adolescents were asked to provide oral informed assent. Interviewers read an information sheet with an explanation of the study purpose and procedures to participants (and parents for 12–14 year olds), requested their verbal informed consent and acknowledged receipt in the tablet before beginning the interviews. Names were not entered into the tablets, nor collected during participatory research activities to preserve anonymity. The lists used to locate the adolescents and all posters developed during the participatory activities were destroyed once data collection was complete.

## Results

The total survey sample size was 444 adolescent girls aged 12–17 and 245 adolescent boys aged 12–17 (Table 1). A total of six groups of adolescents engaged in the participatory research activities, three in each camp: two groups of girls (12–14 years, 15–19 years) and one group of boys (12–14 years in Mile and 15–17 years in Kounoungou). Each group had 12–18 participants.

**Table 1:** Total survey sample size.

Age group and sex	No. of interviews, Kounoungou	No. of interviews, Mile
Girls, 12–14 years	114	110
Girls, 15–17 years	107	113
Boys, 12–14 years	62	70
Boys, 15–17 years	62	51
**Total**	**345**	**344**

### Sociodemographic characteristics

All survey respondents identified as Muslim, and the majority belonged to the Zaghawa (54.0%) ethnic group (Table 2). An overwhelming majority of the respondents had spent ten or more years, or most of their lives, living in the camps (89.5%) and resided with at least one parent. Most adolescents (90.7%) reported living with their mothers, while fewer girls (54.2%) than boys (72.2%) reported living with their fathers. Nearly all adolescents (93.8%) reported being in school at the time of the study, and 44.0% of girls and over half of boys (56.3%) had at least some secondary education. Most adolescents participated in some form of unpaid labor, including household chores, although the types of work differed by sex. The majority of girls prepared meals (56.3%) and/or washed, ironed, or mended clothing (55.4%) without pay. Nearly half of adolescent boys (44.9%) worked in agriculture or livestock, and 34.7% collected firewood or water. Boys (7.3%) were more likely than girls (1.6%) to report no unpaid labor.

**Table 2:** Sociodemographic characteristics, adolescents 12–17 years old, Mile and Kounoungou camps, Chad.

	Total	Girls	Boys	Χ^2^ p-value
	(N = 689) %(n)	(N = 444) %(n)	(N = 245) %(n)	
**Age group**				.43
12–14 years	51.7% (356)	50.5% (224)	53.9% (132)	
15–17 years	48.3% (333)	49.5% (220)	46.1% (113)	
**Ethnic group**				.21
Zaghawa	54.0% (372)	55.6% (247)	51.0% (125)	
Tama	21.9% (151)	22.7% (101)	20.4% (50)	
Djabal	19.4% (134)	17.8% (79)	22.4% (55)	
Other ethnicity	4.6% (32)	3.8% (17)	6.1% (15)	
**Duration lived in the camp**				.11
Less than 10 years	10.5% (72)	12.0% (53)	7.8% (19)	
10 years or more	89.5% (616)	88.0% (390)	92.2% (226)	
**Other adults living in the same house with them**				
Father	60.6% (417)	54.2% (240)	72.2% (177)	< .001
Mother	90.7% (624)	90.3% (400)	91.4% (224)	.66
Other adult family member	55.7% (383)	49.7% (220)	66.5% (163)	< .001
Husband / wife	1.3% (9)	2.0% (9)	0% (0)	.06
Other adult non-family member	0.4% (3)	0.2% (1)	0.8% (2)	.60
**Currently in school**	93.8% (618)	92.7% (396)	95.7% (222)	.18
**Highest level of education completed**				< .001
None	4.7% (32)	3.9% (17)	6.1% (15)	
Some or completed primary school (< 7 years)	46.9% (322)	52.2% (230)	37.6% (92)	
Some or completed secondary school or higher (7 years or more)	48.4% (332)	44.0% (194)	56.3% (138)	
**Unpaid work performed**				
Meal preparation	37.0% (255)	56.3% (250)	2.0% (5)	< .001
Laundry, mending clothes	47.3% (326)	55.4% (246)	32.7% (80)	< .001
House or compound cleaning	35.7% (246)	46.2% (205)	16.7% (41)	< .001
Farming/livestock work	24.1% (166)	12.6% (56)	44.9% (110)	< .001
Firewood or water collection	17.3% (119)	7.7% (34)	34.7% (85)	< .001
Manual labor	18.0% (124)	11.0% (49)	30.6% (75)	< .001
Childcare	16.8% (116)	20.0% (89)	11.0% (27)	.003
Other unpaid work	7.5% (52)	3.6% (16)	14.7% (36)	< .001
Does no unpaid work	3.6% (25)	1.6% (7)	7.3% (18)	< .001

### Sexual and reproductive health knowledge

Girls and boys reported different sources of information on SRH (Table 3). For example, the most important sources of information on puberty for girls were their mother (40.3%) and school (22.3%). The most important sources for boys were school (31.1%) followed by friends (26.2%) and internet/media (24.8%). In contrast, very few girls (1.4%) reported internet or media as an information source. Overall, boys reported better knowledge of menstruation and pregnancy than girls in this survey. Most adolescents knew that menarche indicated a girl’s ability to become pregnant (57.2% of girls, 71.7% of boys) and that a girl could get pregnant the first time she had sex after menarche (57.4% of girls, 74.5% of boys). When asked about early pregnancy, 59.5% of girls and 78.7% of boys knew that early pregnancy was dangerous for the health of the girl and the baby. However, during the participatory research activities, both girls and boys demonstrated a strong understanding of key SRH terms. Boys and girls drew detailed, anatomically accurate female and male reproductive organs, depictions of sexual relationships and abortions, highlighting their foundational SRH knowledge and willingness to engage with SRH educational material. Despite the survey findings that girls were less knowledgeable about menstruation and pregnancy than boys, the girls often cited contraception as an essential resource to maintain good SRH and prevent consequences such as unintended or premarital pregnancy, abortion, dropping out of school, ostracization from their families, and even death. One participant described a girl who gets pregnant at age 15 as one who “*will have difficulties*, *complications during childbirth*. *She may contract diseases*. *She could also die*" (Girl, 12–14 years).

**Table 3:** Sexual and reproductive health knowledge, adolescents 12–17 years old, Mile and Kounoungou camps, Chad.

	Total	Girls	Boys	Χ^2^ p-value
	(N = 689) %(n)	(N = 444) %(n)	(N = 245) %(n)	
**Most important source of information on puberty**				< .001
Mother	26.2% (180)	40.3% (179)	0.4% (1)	
Other family members	14.5% (100)	19.8% (88)	4.9% (12)	
Teacher / school	25.5% (175)	22.3% (99)	31.1% (76)	
Friends	17.0% (117)	11.9% (53)	26.2% (64)	
Internet, radio, TV / film	9.6% (66)	1.4% (6)	24.8% (60)	
Other source	1.2% (8)	0.5% (2)	2.5% (6)	
No source / not informed / don’t know	5.8% (40)	3.8% (17)	9.4% (23)	
**The onset of menstruation means that girls have the ability to become pregnant.**				< .001
True*	62.4% (429)	57.2% (254)	71.7% (175)	
False	27.5% (189)	32.9% (146)	17.6% (43)	
Don’t know	10.2% (70)	9.9% (44)	10.7% (26)	
**A girl can get pregnant the first time she has sex.**				< .001
True*	63.% (436)	57.4% (255)	74.5% (181)	
False	27.2% (187)	31.5% (140)	19.3% (47)	
Don’t know	9.3% (64)	11.0% (49)	6.2% (15)	
**Getting pregnant early in puberty can be bad for the health of the girl and the baby.**				< .001
True*	66.3% (456)	59.5% (264)	78.7% (192)	
False	23.4% (161)	30.9% (137)	9.8% (24)	
Don’t know	10.3% (71)	9.7% (43)	11.5% (28)	
**Using a condom can protect against pregnancy.**				< .001
True*	47.7% (327)	38.4% (170)	64.9% (157)	
False	32.8% (225)	43.1% (191)	14.0% (34)	
Don’t know	19.4% (133)	18.5% (82)	21.1% (51)	
**A girl can use herbs to protect herself from pregnancy.**				< .001
True	29.7% (202)	35.1% (154)	19.8% (48)	
False*	53.6% (365)	52.2% (229)	56.2% (136)	
Don’t know	16.7% (114)	12.8% (56)	24.0% (58)	
**Knowledge of modern contraceptive methods (prompted)**				.18
Knows no modern methods	30.9% (213)	29.1% (129)	34.3% (84)	
Knows at least one modern method	69.1% (476)	70.9% (315)	65.7% (161)	
Tubal ligation	9.6% (66)	13.1% (58)	3.3% (8)	< .001
Vasectomy	8.1% (56)	9.9% (44)	4.9% (12)	.03
Intra-uterine device (IUD)	13.9% (96)	16.7% (74)	9.0% (22)	.007
Implants	19.9% (137)	20.7% (92)	18.4% (45)	.52
Injectables	50.9% (351)	52.7% (234)	47.8% (117)	.24
Contraceptive pills	55.4% (382)	61.7% (274)	44.1% (108)	< .001
Condoms	39.5% (272)	29.1% (129)	58.4% (143)	< .001
Emergency contraception	16.7% (115)	21.2% (94)	8.6% (21)	< .001
Traditional method	23.9% (165)	30.4% (135)	12.2% (30)	< .001

*Indicates correct response to knowledge questions

Most boys (64.9%) knew that condoms could prevent pregnancy, compared to only 38.4% of girls although many (18.5% girls, 21.1% boys) were unsure. More than half of girls (52.2%) and boys (56.2%) knew that using herbs or plants would not prevent pregnancy, but 12.8% of girls and 24.0% of boys were unsure. Over two thirds (69.1%) of adolescents had heard of at least one modern contraceptive method. Even when prompted, 30.9% of adolescents were unable to identify any modern contraceptive method. Girls’ knowledge of contraceptive methods was better than boys for all methods except condoms, which boys were more likely to know, and implants and injectables, where knowledge was similar. Long-acting reversible (IUD, implant) and permanent (tubal ligation, vasectomy) methods were the least known methods.

### Access to contraception

Approximately half of girls (49.9%) and 32% of boys thought an adolescent in their neighborhood would know where to seek contraception (Table 4). Most reported they would seek contraception at the health center (62.7% of girls, 75.2% of boys), and more girls than boys mentioned a pharmacy (27.2% of girls, 17.4% of boys). Trust in the confidentiality of contraceptive services varied considerably among adolescents. Although 58.2% of girls and 54.8% of boys reported that the health worker would maintain confidentiality if they sought a contraceptive method, 34.1% of girls and 22.4% of boys thought that they would inform their parents. During participatory activities, adolescents highlighted additional barriers including long travel times to the health center and a widespread lack of transportation in the camps.

**Table 4:** Access to contraception, adolescents 12–17 years old, Mile and Kounoungou camps, Chad.

	Total	Girls	Boys	Χ^2^ p-value
	(N = 689) %(n)	(N = 444) %(n)	(N = 245) %(n)	
**An adolescent in your neighborhood who needs a contraceptive method knows where to go to get one.**				< .001
Yes	40.5% (278)	38.1% (169)	44.7% (109)	
No	43.5% (299)	49.9% (221)	32.0% (78)	
Don’t know	16.0% (110)	12.0% (53)	23.4% (57)	
**Where adolescents go for contraception**				< .001
Health center	67.6% (188)	62.7% (106)	75.2% (82)	
Pharmacy	23.4% (65)	27.2% (46)	17.4% (19)	
Traditional healer	5.0% (14)	8.3% (14)	0% (0)	
Other	4.0% (11)	1.8% (3)	7.3% (8)	
**Trust in the confidentiality of contraceptive services at the health center. The health worker would…**				< .001
…Keep it a secret	57.0% (390)	58.2% (258)	54.8% (132)	
…Tell his/her parents	30.0% (205)	34.1% (151)	22.4% (54)	
Don’t know	13.0% (89)	7.7% (34)	22.8% (55)	

### Attitudes relating to SRH

In the survey, few adolescents agreed that adolescents may have sex before marriage (Table 5). The participatory research activities, however, revealed more nuanced attitudes. Adolescents occasionally added premarital sex, including rape, on the boys’ social and developmental timelines they created in small groups. One female participant in Mile explained that *"A man is a man*, *he can have sex*, *but no one is disgusted by this*. *Because he is a man*.*"* (Girl, 15–19 years old). Adolescents frequently indicated that girls have premarital sex by listing unwanted pregnancy, rape, and/or abortion as negative events on the timelines earlier than marriage, forced marriage, or early marriage.

**Table 5:** Attitudes relating to sexual and reproductive health, adolescents 12–17 years old, Mile and Kounoungou camps, Chad.

	Total	Girls	Boys	Χ^2^ p-value
	(N = 689) %(n)	(N = 444) %(n)	(N = 245) %(n)	
**Girls may have sex before they marry.** *(Agree)*	17.7% (121)	17.5% (77)	18.1% (44)	.94
**Boys may have sex before they marry.** *(Agree)*	22.9% (157)	22.4% (99)	23.9% (58)	.73
**A girl or woman who uses family planning has many sexual partners.**† *(Disagree)*	66.3% (413)	73.3% (316)	50.5% (97)	< .001
**It would be too embarrassing for someone like me to get family planning.**† *(Disagree)*	56.9% (359)	67.3% (294)	33.5% (65)	< .001
**If I (my partner) got pregnant when I (she) didn’t want to be, I may try to (help her) terminate the pregnancy.**† *(Agree)*	12.4% (83)	14.3% (63)	8.8% (20)	.06
**I know someone in the camp who has terminated a pregnancy.**	38.1% (261)	38.6% (171)	37.2% (90)	.93

†Variable with missing data >5% and <10%.

Consistent with their better knowledge, girls held more favorable attitudes towards contraception than boys, for example 73.3% of girls and 50.5% boys disagreed that a girl or woman using contraception has multiple sexual partners. Two thirds of girls (67.3%) but only 33.5% of boys disagreed that seeking contraception would be too embarrassing for someone like them. This aligns with girls’ discussions during the participatory research activities of the comparatively greater consequences of a premarital pregnancy. In the words of one female participant, *"For a girl who is not married*, *it’s a mess*, *it’s very complicated*. *If she gets pregnant*, *it’s hard for her*. *The community stigmatizes her*, *her mother insults her and her father turns his back on her*. *She is alone and doesn’t know what to do or where to go to give birth”* (Girl, 15–19 years old). Contraception surfaced during the boys’ participatory research activities as well. For both boys’ groups, condoms were ranked in the top three most important resources that boys needed to maintain good SRH.

Despite girls’ more favorable attitudes toward contraception, they responded similarly to boys when asked about seeking abortion. Only 12.4% of all adolescents agreed that they may try to end an unwanted pregnancy (or help their partner to do so) if needed. However, 38.1% of adolescents knew someone in the camp who terminated a pregnancy. The participatory research activities revealed that some boys and girls view abortion as an essential means to prevent the stigma and social isolation of an unintended, premarital pregnancy.

“*Everyone was badmouthing me behind my back when I got pregnant [while unmarried]*, *whether in the street or in the neighborhood with my girlfriends*. *It’s hard to go through all that*. *I decided to take pills to end the pregnancy*" (Girl, 12–14 years old).

Participant 1: “For me, if I get pregnant, I will take pills to end the pregnancy. It’s simple.”

*Participant 2*: *“Because when we get pregnant outside of marriage*, *even our girlfriends leave us*, *they fear us*." (Girls, 12–14 years old)

### Romantic relationships

Only 5% of girls and 0.8% of boys were married, and 11.1% of unmarried girls and 4.6% of unmarried boys had already received a marriage proposal (Table 6). This is consistent with the timelines developed in the participatory research where both boys and girls placed marriage later on the boys’ timelines than on the girls’ timelines. Among unmarried adolescents, 17.6% reported having had a boyfriend or girlfriend, of which most (95.8% of girls, 82.2% of boys) reported currently having one. Only 11.5% reported having already had sex themselves, while larger proportions (24.3% of girls, 16.7% of boys) reported that at least some of their close friends had already had sex.

**Table 6:** Romantic relationships, adolescents 12–17 years old, Mile and Kounoungou camps, Chad.

	Total	Girls	Boys	Χ^2^ p-value
	(N = 689) %(n)	(N = 444) %(n)	(N = 245) %(n)	
**Currently married**	3.5% (24)	5.0% (22)	0.8% (2)	.01
**Already received a marriage proposal**	8.7% (58)	11.1% (47)	4.6% (11)	.01
**Ever had a boyfriend / girlfriend**	17.6% (117)	17.1% (72)	18.5% (45)	.71
**Currently have a boyfriend / girlfriend** *(N = 117)*	90.6% (106)	95.8% (69)	82.2% (37)	.03
**Ever had sex**	11.4% (78)	10.8% (48)	12.6% (30)	.49
**Number of close friends they think have already had sex**				.002
None of my close friends	65.7% (449)	61.0% (271)	74.0% (178)	
At least some of my close friends	21.7% (148)	24.3% (108)	16.7% (40)	
Don’t know	12.6% (86)	14.6% (65)	8.8% (21)	
**Reasons for not having sex**				
I don’t feel ready / am too young	60.8% (366)	62.8% (247)	56.9% (119)	.23
My parents would be very angry	29.4% (177)	34.1% (134)	20.6% (43)	< .001
It’s against my culture / religion	35.7% (215)	23.9% (94)	57.9% (121)	< .001
I don’t think you should have sex before marriage	17.3% (104)	18.6% (73)	14.8% (31)	.32
Fear of unwanted pregnancy or getting a sexually transmitted infection	12.3% (74)	11.2% (44)	14.4% (30)	.30
Haven’t had a boyfriend / girlfriend or the opportunity to have sex	6.1% (37)	7.1% (28)	4.3% (9)	.24

Among girls who reported not having had sex, the primary reasons included feeling too young or not ready (62.8%) and fear of angering their parents (34.1%). For the boys, the primary reasons for abstaining were that premarital sex was against their religion (57.9%), and they were too young or not ready (56.9%). Most of these adolescents (82.5% of girls, 91.8% of boys) reported planning to wait until marriage to have sex.

### Sexual activity

The mean age of sexual debut was 15 years among girls and boys who had ever had sex. Although most adolescents (56.5% of girls, 73.3% of boys) reported that their first sexual partner was a boyfriend or girlfriend, many girls (37.0%), but only two boys, had sex for the first time with a spouse (Table 7). While 56.5% of girls reported their first sexual partner was three or more years older, all boys reported their partner was approximately the same age as them (p < .001). The majority (65.2% of girls,100% of boys) reported that the decision to have sex was made jointly by both partners. However, 21.7% of girls reported that the boy forced or persuaded them to have sex. Considerably more sexually active girls (32.6%) than boys (10.9%) reporting having ever used a contraceptive method to prevent pregnancy. For example, only 23.9% of girls and 10.0% of boys reported that they used a condom the last time they had sex, and 28.3% of girls and none of the boys reported current contraceptive use. The number of current users was small: 11 reported using injectables and two reported using pills. No differences in contraceptive use by marital status were noted.

**Table 7:** Sexual activity*, adolescents 12–17 years old, Mile and Kounoungou camps, Chad.

	Total	Girls	Boys	p-value
	(N = 79) %(n)	(N = 48) %(n)	(N = 30) %(n)	
**Age at first sexual intercourse**				
10–14 years	40.0% (30)	40.0% (18)	40.0% (12)	1.0
15–17 years	60.0% (45)	60.0% (27)	60.0% (18)	
**Relationship to the first person with whom they had sex**				.006
Husband / wife	25.0% (19)	37.0% (17)	6.7% (2)	
Boyfriend / girlfriend	63.2% (48)	56.5% (26)	73.3% (22)	
Another person	11.8% (9)	6.5% (3)	20.0% (6)	
**Decision to have sex for the first time**				.001
Girl forced / persuaded boy to have sex	7.9% (6)	13.0% (6)	0% (0)	
Boy forced / persuaded girl to have sex	13.2% (10)	21.7% (10)	0% (0)	
Both equally willing	78.9% (60)	65.2% (30)	100.0% (30)	
**Used a condom the last time they had sex**	18.4% (14)	23.9% (11)	10.0% (3)	.13
**Ever used a contraceptive method**	23.7% (18)	32.6% (15)	10.0% (3)	.02
**Currently using a contraceptive method**	17.1% (13)	28.3% (13)	0%	.004

*Limited to adolescents who reported ever having had sex

## Discussion

This study is among the first to assess SRH knowledge, attitudes, and behaviors among adolescent refugees in Chad. It is also one of the few studies on adolescent SRH in humanitarian settings that includes boys as well as girls.

### SRH knowledge

Overall, adolescents’ SRH knowledge was low, consistent with findings on SRH knowledge among adolescents and adolescent refugees in other low- and middle-income countries [17, 18]. For example, our findings were at the lower end of the range in a Guttmacher Institute study finding that 40–60% of adolescents in 13 sub-Saharan African countries knew where to seek contraception in 2015 [17]. Our survey results suggest that boys have better SRH knowledge than girls, even for topics like menstruation and pregnancy, but worse knowledge of contraception. However, the participatory research findings illustrate that girls do indeed have foundational SRH knowledge. This discrepancy may be a result of girls being more shy or uncomfortable discussing SRH topics with a stranger during an interview, rather than amongst their peers in small groups during the participatory activities. While both girls and boys reported that school is a good source of information on puberty, girls were more likely to report learning about this from their mothers. Information from mothers may be less accurate or girls may be embarrassed to discuss this information at home. Boys reported friends and internet/media as common sources of information similar to findings elsewhere [19]. Although this study did not directly ask about mobile phone or internet access or use, boys generally are twice as likely as girls to have access to mobile phones or internet, including in Chad [14, 20]. However, the quality of the information boys access is unknown. The ProGeSan program provided SRH training to teachers in the camps’ schools as well as to peer educators who organize education sessions in the schools on adolescent SRH and prevention of sexually transmitted infections. In addition, they implemented the Girl Shine program, a life skills program with cohorts of adolescent girls in the camps; however, only a few girls in our survey had participated in this program.

This survey also exposed a knowledge-behavior gap among boys, who had comparatively greater knowledge of condoms than girls but reported considerably lower condom use. This gap may be partially explained by boys’ limited knowledge of where to seek condoms and boys’ higher level of shame associated with seeking contraception. Girls were more likely than boys to report an older sexual partner, and older boys may have greater knowledge of where to find condoms and greater agency to use them. Other possible explanations include provider and community stigma toward contraceptive use by adolescent boys, boys’ embarrassment at seeking condoms in the maternity (where condoms are available for free in the camps), or condom cost and availability elsewhere in the camps [21, 22]. Increasing the number and types of places where adolescents can seek condoms and other contraceptives would make contraceptive service delivery more adolescent-responsive and improve access.

Despite the availability of long-acting reversible contraceptives (LARC) at the camp health centers, LARC were both the least known and least used modern methods among adolescents. Although adolescent girls in conflict-affected areas of Uganda and the Democratic Republic of the Congo have reported LARC use, short-acting contraceptives are more common among adolescent and young women in Sub-Saharan Africa [23–25]. Global evidence suggests that LARC represent an untapped potential strategy for reducing unintended pregnancies among adolescents [26, 27]. LARC are highly effective in preventing pregnancy, and they minimize the time spent traveling to and potentially being seen at the health center, a major concern for many adolescents [21, 28].

### SRH attitudes

Adolescent attitudes toward SRH and contraception were mixed, although girls tended to hold more favorable views toward contraception than boys. These attitudes highlight the stigma associated with premarital sex and pregnancy that are commonly found among adolescents in stable settings [22, 29]. Although most adolescents reported feeling too young to engage in sexual relations, boys were more likely to report religious prohibitions for abstaining whereas girls were more likely to report fear of parental anger. The participatory findings provided a rich picture of the social consequences unmarried girls face when they become pregnant. The comparatively more severe consequences experienced by girls may be a driver for their increased contraceptive use and explain their more favorable attitudes toward contraception. Generally, adolescents held unfavorable attitudes toward abortion, a not uncommon attitude among adolescents in low and middle income countries [19, 30]. Nonetheless, abortion was cited frequently in the participatory activities as a negative event in the life of a typical girl in the camps, and many adolescents reported knowing someone in the camp who had an abortion. While adolescents mentioned the use of pills to end a pregnancy, we were unable to confirm the type of pills they referred to.

### Romantic relationships and sexual activity

Early marriage was uncommon in this population, despite findings in other humanitarian settings which report increases in early marriage [18]. This may be due to girls’ high level of education in this population, given the well-documented association between low education and early marriage [31, 32]. However, girls were considerably more likely than boys to be married. The participatory research activities revealed girls’ fears of early marriage and pregnancy, which were described as threats to their education and health, similar to adolescent girls in other humanitarian settings [18, 33]. The majority of sexually active adolescents reported that their first sexual partner was a non-marital partner. This finding, despite the stigma around premarital sex in this community, underscores the importance of ensuring SRH services, especially contraceptive services, are acceptable and accessible to unmarried adolescent girls and boys. Despite the boys’ low reported condom use, the boys in the participatory research activities highlighted condoms as one of their top resources needed to maintain good SRH suggesting a need to make condoms more accessible to them. Condoms should be made available for free in locations outside of the maternity where boys may be embarrassed to go. Given the low condom knowledge, among girls in particular, information on condoms should be made available to girls and boys. In addition, education should also focus on negotiation skills regarding contraceptive and condom use–for both girls and boys.

While 11% of adolescents reported sexual activity, this may be underreported partially due to social desirability bias, given that adolescent sexual activity and premarital pregnancies are highly stigmatized in this setting, like in many others [19, 34]. Adolescents’ estimates of the level of sexual activity amongst their close friends were considerably higher than what they reported for themselves. Although contraceptive use among sexually active boys was near zero, it is possible that some boys were unaware that their partners were indeed using contraception but had not disclosed it to them. Despite low contraceptive knowledge and barriers to contraceptive access, both unmarried and married sexually active girls reported relatively high levels of contraceptive use compared to contraceptive prevalence among adolescents in other sub-Saharan African countries [23, 35, 36]. In many communities, including this one, boys and men play important roles in decision-making around contraceptive use [37–39]. The very low contraceptive use and limited knowledge of contraceptive methods reported here by boys is concerning given their role as potential future husbands. Engaging boys in gender-transformative SRH programming is critical to support girls’ and women’s access to contraceptive services.

### Limitations

While all research participants were reminded of the survey’s confidentiality, some participants may not have been comfortable disclosing information about their sexual activity due to social desirability bias, generalized SRH stigma or a prior negative sexual experience [40]. Although the interviewers discussed during training how to translate each question, it is possible some used different wording for some questions in the field. Interviewers made concerted efforts to reach the adolescents who were randomly sampled for the survey, including visiting the adolescents’ homes multiple times or seeking them elsewhere in the camp, but some were unreachable and replaced by randomly selected peers in the same age cohort who lived in the same or a nearby zone of the camp. It is unknown if those who could not be found were different from those who were. During small group work in the participatory research, adolescents sometimes discussed in local languages that not all of our research facilitators could speak, meaning that some detail may have been missed by the research teams.

## Conclusions

This study demonstrates the need to make existing SRH services more adolescent-responsive in humanitarian settings. Adolescents–married and unmarried—should be engaged in an age-appropriate, meaningful way so that their experiences and voices are included in the planning and designing of SRH services [41]. Although SRH programs and research typically prioritize girls, it is also important to address boy’s experiences, needs, and barriers to accessing SRH services. Barriers to accessing SRH services should be minimized to increase service acceptability and accessibility among adolescents [21]. This includes addressing community stigma surrounding adolescents’ use of SRH services and improving adolescents’ trust in the confidentiality of SRH services in the camps. Efforts to reduce community stigma must include both adolescents and adult community members and should incorporate strategies from gender-transformative approaches and collaboration with other sectors, including the education and protection sectors. Multi-level interventions that include the provision of adolescent-responsive services and address demand-side barriers at the individual, caregiver, and community level are critical to create an enabling environment that is supportive of adolescent needs.

## Supporting information

S1 Checklist Inclusivity in global research. (DOCX)
